# Diet‐induced obesity in mice reduces placental efficiency and inhibits placental mTOR signaling

**DOI:** 10.1002/phy2.242

**Published:** 2014-02-26

**Authors:** Susanne Lager, Anne‐Maj Samulesson, Paul D. Taylor, Lucilla Poston, Theresa L. Powell, Thomas Jansson

**Affiliations:** 1 Department of Obstetrics and Gynecology, Center for Pregnancy and Newborn Research, University of Texas Health Science Center, San Antonio, Texas; 2 Division of Women's Health, Women's Health Academic Centre, King's College London and King's Health Partners, London, UK

**Keywords:** Cell signaling, inflammation, insulin, pregnancy

## Abstract

As in humans, obesity during pregnancy in mice results in elevated maternal insulin levels and metabolic programming of offspring. mTOR signaling regulates amino acid transport and may function as a placental nutrient sensor. Because obesity is a condition with increased nutrient availability, we hypothesized that diet‐induced obesity activates placental mTOR signaling. To test this hypothesis, female C57BL/6J mice were fed an obesogenic diet or standard chow prior to and throughout pregnancy. Fetuses and placentas were collected at gestational day 18. Using Western blot analysis, placental mTOR activity was determined along with energy, inflammatory, and insulin signaling pathways (upstream modulators of mTOR). At gestational day 18, fetal and placental weights did not differ, however, in obese dams, the fetal/placental weight ratio was lower (*P *<**0.01). In placentas from obese dams, mTOR signaling was inhibited, as determined by decreased Rheb and S6K1 expression, and lower rpS6 phosphorylation (*P *<**0.05). In contrast, energy, inflammatory, and insulin signaling pathways were unaffected. Contrary to our hypothesis, diet‐induced obesity in pregnant mice was associated with inhibition of placental mTOR signaling. However, this finding is consistent with the lower fetal/placental weight ratio, indicating reduced placental efficiency.

## Abstract (precis)

In this report, we explore placental signaling in an obese mouse model known to result in adverse metabolic programming of the offspring without altering fetal growth. This model parallels with human pregnancies, where maternal obesity also is associated with adverse metabolic programming. In this report, we show that maternal obesity in the mouse leads to reduced placental efficiency potentially caused by lowered signaling activity of the mTOR pathway (a known positive regulator of placental nutrient transport) and that the placentas of obese, hyperinsulinemic dams are insulin resistant.

## Introduction

Many women enter pregnancy overweight or obese (Chu et al. 2009; Wallace et al. 2012). Maternal obesity during pregnancy is associated with an increased risk of pregnancy complications, as well as increased birth weight (Sebire et al. 2001). Being large at birth is associated with an elevated risk of developing metabolic health disorders later in life (Boney et al. 2005; Evagelidou et al. 2006; Sparano et al. 2012). Furthermore, children born to obese mothers are more likely to become obese themselves (Whitaker 2004).

In a well‐characterized and extensively studied mouse model, females are fed a high‐fat, high‐sugar diet prior to mating resulting in obesity before pregnancy (Samuelsson et al. 2008). In this model, maternal obesity results in adverse metabolic programming of the offspring, which as adults have an elevated body weight and fat mass (Samuelsson et al. 2008). The mechanisms linking obesity in pregnancy to altered fetal development and programming of adult disease remain to be fully established, but there is an increasing awareness that placental responses to changes in the maternal environment may contribute (Jansson and Powell 2007; Jansson et al. 2012). It has previously been reported that in late pregnancy, these obese dams have elevated serum insulin and leptin levels, but normal blood glucose, consistent with insulin resistance (Samuelsson et al. 2008); however, the impact of maternal obesity on the placenta have not been examined. Therefore, the aim of this study was to investigate the effects of maternal obesity on placental signaling pathways.

The mechanistic Target of Rapamycin (mTOR) signaling pathway has been proposed to function as a nutrient sensor in the placenta (Jansson and Powell 2006; Jansson et al. 2012), matching fetal growth to maternal nutrient availability by altering placental growth and nutrient transport. Placental mTOR activity is positively correlated to fetal growth in animal models (Rosario et al. 2011) and in humans (Roos et al. 2007; Gaccioli et al. 2011; Jansson et al. 2013). mTOR signaling activity is commonly assessed by phosphorylation of down‐stream targets S6 Kinase 1 (S6K1), ribosomal protein S6 (rpS6), and eukaryotic translation initiation factor 4E‐binding protein 1 (4EBP1). Numerous factors modulate mTOR activity, including amino acids, cellular energy levels, and growth factors (Laplante and Sabatini 2012). Many of these stimuli impinge on the tuberous sclerosis complex (TSC)1‐TSC2 heterodimer, located upstream of mTOR (Duran and Hall 2012). The TCS1‐TSC2 complex inhibits the protein: ras homolog enriched in brain (Rheb), which is a positive regulator of mTOR (Duran and Hall 2012).

Maternal obesity in animal models (Samuelsson et al. 2008) and humans (Challier et al. 2008; Catalano et al. 2009) is associated with elevated circulating levels of insulin, which activates mTOR (Laplante and Sabatini 2012). In cultured primary human trophoblast cells (PHTs) insulin stimulate amino acid uptake mediated by an mTOR‐dependent mechanism (Roos et al. 2009a,b. Obese pregnant women also have elevated circulating levels of inflammatory markers (Challier et al. 2008; Roberts et al. 2011) such as interleukin (IL)‐6, which stimulates amino acid uptake in PHTs (Jones et al. 2009). Inflammatory signals can also increase mTOR signaling activity (Lee et al. 2007).

Therefore, based on this evidence, we tested the hypothesis that diet‐induced obesity in pregnant mice activates the placental mTOR signaling pathway.

## Methods

### Animals

Female C57BL/6J mice were fed an obesogenic diet or standard chow for 6 weeks prior to mating and throughout pregnancy as previously described (Samuelsson et al. 2008). Maternal plasma levels of cholesterol, glucose, insulin, leptin, and triglycerides from these dams have previously been published (Samuelsson et al. 2008); the data presented in this article have not been reported previously. The standard chow diet consisted of 7% simple sugars, 3% fat, 50% polysaccharide, and 15% protein [w/w] (RM1, Special Dietary Services, Essex, UK; energy 3.5 kcal/g; *n* = 9). The obesogenic diet consisted of pellets (10% simple sugars, 20% animal lard, 28% polysaccharide, and 23% protein [w/w], Special Dietary Services, energy 4.5 kcal/g) and ad libitum access to sweetened condensed milk (approx. 55% simple sugar, 8% fat, and 8% protein, [w/w], Nestle). The milk was supplemented with micronutrient mineral mix (AIN93G; Special Dietary Services). The macronutrient and calorific intake of the obesogenic diet was estimated to approx. 16% fat, 33% simple sugars, 15% protein (energy 4.0 kcal/g; *n* = 10) based on daily measurements of intake of pellets and milk. The appearance of a copulation plug was assigned as gestational day 0. The dams were euthanized on gestational day 18. Maternal nonfasting blood was collected by cardiac puncture and centrifuged (15 min, 2000 g) for plasma analysis. Fetal and placental weights and fetal lengths were recorded. Plasma and placental tissue were frozen and stored at −80°C until further processing.

### Western blot

Placental tissue from each litter were pooled and homogenized on ice in buffer D (250 mmol/L sucrose, 10 mmol/L hepes, pH 7.4 at +4°C) containing protease and phosphatase inhibitors (Sigma‐Aldrich, St. Louis, MO). The homogenized tissue was centrifuged for 15 min at 16,000 g, 4°C. The supernatant was collected and protein concentrations determined with a BCA kit (Thermo Scientific, Rockford, IL). Proteins were separated using precast gels from BioRad (Hercules, CA) and transferred to polyvinyl difluoride (PVDF) membranes. The membranes were stained for total protein with Amido Black Stain (Sigma‐Aldrich) for 1 min followed by three washes with destaining solution (50% methanol, 7% acetic acid) for 2 min (Lanoix et al. 2012). Membranes were blocked in 5% milk in tris‐buffered saline with 0.1% tween (TBS‐T) for 1 h. Membranes were incubated with primary antibody overnight at 4°C. Primary antibodies were purchased from Cell Signaling Technology (Davers, MA): 4EBP1 (catalog #9452, #9459 and #9455), AMP‐activated protein kinase (AMPK; #2532 and #2535), AKT (#9275, #4056 and #4060), extracellular‐signal‐regulated kinase (ERK1/2; #4695 and #4370), growth factor receptor‐bound protein (GRB2; #3972), inhibitor of nuclear factor ĸ B (IĸB; #4812), c‐Jun N‐terminal kinases/stress‐activated protein kinase (JNK; #9252 and #4668), mitogen activated protein kinase (p38 MAPK; #9212), phosphoinositide 3‐kinase (PI3K p85; #4292), rpS6 (#2217 and #4858), Rheb (#4935), S6K1 (#9202 and #9205). Primary antibody targeting phosphorylated p38 MAPK (ab32557) was purchased from Abcam (Cambridge, MA), phosphorylated‐insulin receptor substrate‐1 (IRS‐1; I2658) from Sigma, and total IRS1 (06‐248) from Millipore (Billerica, MA). After washing with TBS‐T, membranes were incubated with appropriate peroxidase‐labeled IgG antibody (Cell Signaling Technology) for 1 h at room temperature. Immunolabeling was detected with SuperSignal Dura West or ECL detection solution (Thermo Scientific). Relative density of bands was measured with ImageJ software (National Institutes of Health). Target protein expressions were adjusted for amount of total protein (Amido Black stain) to ensure equal loading. The mean value of controls was arbitrarily assigned a value of 1.0 for comparisons between groups.

### Plasma corticosterone analysis

The plasma corticosterone concentration was analyzed with a commercial ELISA kit (EIA5186; DRG Instruments, Marburg, Germany) in accordance with the manufacturer's instructions. Each sample was analyzed in duplicate.

### Data presentation and statistics

The data are presented as mean ± SEM. Statistical analysis was carried out using GraphPad Prism 5 (version 5.04; GraphPad Software, La Jolla, CA). Differences between the two groups were evaluated by unpaired *t*‐test. *P*‐value < 0.05 was considered significant.

## Results

### Maternal obesity reduces the fetal/placental weight ratio

There were no significant differences in litter size, litter weight, fetal weight, fetal length, or placental weight between control and obese dams (Table 1). However, the fetal/placental weight ratio was significantly lower in obese dams (−23%, *P *<**0.01; Table 1), suggesting a reduced placental efficiency in these pregnancies.

**Table 1.** Fetal and placental characteristics.

	Control (*n* = 9)	Obese (*n* = 9)
Litter size (number of fetuses)	7.7 ± 0.4	8.1 ± 0.6
Litter weight (g)	7.83 ± 0.57	7.21 ± 0.80
Average fetal weight (g)	1.07 ± 0.07	0.92 ± 0.05
Average fetal length (cm)	2.08 ± 0.07	1.94 ± 0.06
Average placental weight (g)	0.09 ± 0.005	0.11 ± 0.006
Fetal weight/placental weight ratio	11.34 ± 0.55	8.70 ± 0.66^**^

Data are presented as mean ± SEM. **P < 0.01, versus control (unpaired *t*‐test).

### mTOR signaling is reduced in placentas from obese dams

**Figure 1.**
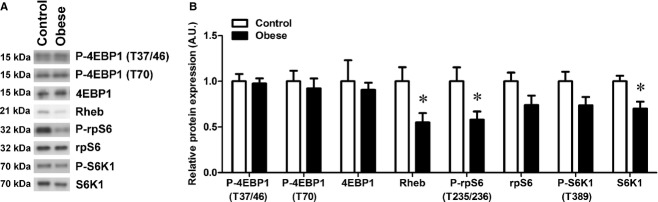
Inhibition of mTOR signaling in placentas from obese dams. Western blots of phosphorylation (P) and/or expression of 4EBP1, Rheb, rpS6, and S6K1 in placental homogenates from control and obese dams collected at gestational day 18 (A). After normalization to total protein stain (Amido Black), the mean density of control samples was assigned a value of 1; summarized Western blot results shown in histogram (B). Data are presented as mean + SEM. *P < 0.05, *t*‐test; Control, *n* = 5; Obese, *n* = 7.

Maternal obesity in pregnant mice reduced placental mTOR signaling, as determined by phosphorylation of rpS6 (−42%, *P *<**0.05; Figure 1). Furthermore, expression of S6K1 (which phosphorylates rpS6) and Rheb (upstream activator of mTOR) were significantly reduced in placentas from obese dams (−30% and −45%, respectively, *P *<**0.05; Figure 1). Expression and phosphorylation of 4EBP1 were not affected by maternal obesity (Figure 1).

### Placental energy, inflammatory, and insulin signaling pathways are not affected by maternal obesity

**Figure 2.**
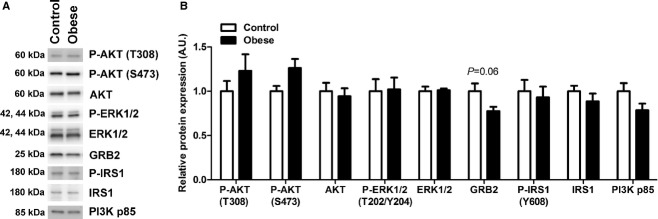
Placental insulin signaling is not affected by maternal obesity. Western blots of phosphorylation (P) and/or expression of AKT, ERK1/2, GRB2, IRS1, and PI3K p85 in placental homogenates from control and obese dams (A). After normalization to total protein stain (Amido Black), the mean density of control samples was assigned a value of 1. The summarized Western blot data are shown in histogram (B). Data are presented as mean + SEM. Control, *n* = 5; Obese, *n* = 7.

**Figure 3.**
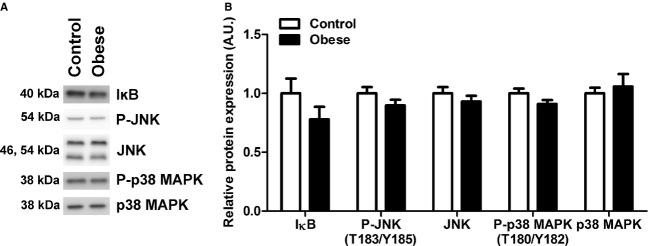
Placental inflammation is not affected by maternal obesity. Western blots of phosphorylation (P) and/or expression of IĸB, JNK, and p38 MAPK in placental homogenates from control and obese dams (A). After normalization to total protein stain (Amido Black), the mean density of control samples was assigned a value of 1; summarized Western blot results shown in histogram (B). Data are presented as mean + SEM. Control, *n* = 5; Obese, *n* = 7.

In order to explore possible mechanism underlying inhibition of placental mTOR signaling in obesity, we examined the activation of energy, insulin, and inflammatory pathways, which are upstream modulators of mTOR. Cellular energy status did not differ significantly between control and obese placentas as measured by AMPK phosphorylation (Control: 1.0 ± 0.2 vs. Obese: 0.7 ± 0.1, arbitrary units) and AMPK total expression (Control: 1.0 ± 0.06 vs. Obese: 0.96 ± 0.10, arbitrary units). Furthermore, maternal obesity in mice had no effect on placental insulin signaling activity as measured by phosphorylation of AKT (T308, S473), ERK1/2 (T202/Y204), and IRS1 (Y608) (Figure 2). Similarly, placental total expression of AKT, ERK1/2, GRB2, IRS1, and PI3K p85 did not significantly differ between control and obese dams (Figure 2). The expression of GRB2 trended toward a lower expression in placentas of the obese dams, but did not reach statistical significance (*P *=**0.06). Lastly, maternal obesity did not alter the expression or activation of placental inflammatory signaling pathways: I*κ*B, JNK, and p38 MAPK (Figure 3).

### Maternal corticosterone levels

At gestational day 18, maternal plasma corticosterone levels were on average 28% higher in the obese dams (219.3 ± 32.0 nmol/L; *n* = 10) compared to the control dams (172.0 ± 23.9 nmol/L; *n* = 7), however the difference was not statistically significant.

## Discussion

In this study, we show that the mTOR signaling pathway in placenta is influenced by maternal obesity in pregnant mice. However, in contrast to our hypothesis, diet‐induced obesity was associated with an inhibition of placental mTOR signaling. Yet, this finding is consistent with the lower fetal/placental weight ratio, suggesting a reduced placental efficiency.

Although obese women are more likely to deliver large‐for‐gestational‐age (LGA) babies,, the majority of children born to obese mothers are not LGA (Ehrenberg et al. 2004) and some demonstrate fetal growth restriction, particularly in association with pre‐eclampsia. There is evidence that children born to obese mothers (independent of birth weight) are at increased risk of becoming obese themselves (Whitaker 2004). Furthermore, Catalano and coworkers have shown that body composition and insulin sensitivity differs between similar‐sized babies born to normal weight or obese mothers (Catalano et al. 2009). These findings suggest that adverse metabolic programming is not limited to infants with altered fetal growth, but also occurs within the normal range of fetal growth. These findings are consistent with observations from our mouse model of diet‐induced maternal obesity, in which offspring are metabolically programmed despite largely normal fetal growth (Samuelsson et al. 2008). In this study, we show that neither fetal nor placental weights are significantly altered by maternal obesity, yet the placental efficiency is reduced and placental signaling affected. These changes may affect the amount and/or composition of nutrients being transported to the fetus, altering the intrauterine environment enough to result in adverse metabolic programming without affecting fetal growth.

In our mouse model, multiple components of the mTOR signaling pathway were affected by diet‐induced maternal obesity. The reduced total expression of S6K1 and Rheb is consistent with a reduced capacity to transmit activating signals, and the lower expression could potentially become a rate‐limiting step. Indeed, down‐stream of S6K1 the activation (measured by phosphorylation) of rpS6 was significantly reduced in placentas from obese dams. This reduced mTOR signaling activity could result in decreased transport of nutrient across the placenta. Fetal growth is dependent on nutrient availability, and, albeit not yet assessed in this mouse model of maternal obesity, we speculate that placental nutrient transport capacity may be reduced because mTOR is a known positive regulator of trophoblast amino acid transport (Roos et al. 2009a,b. A decreased‐transport capacity per gram placenta is also consistent with the reduction in fetal/placental weight ratio. A limitation of this study was the focus on gene and protein expression relevant to the mTOR signaling, and the absence of the direct measurement of transplacental transport of amino acids and glucose or expression of transporter proteins. Studies are now warranted to determine whether the next flux of nutrients is indeed compromised.

To identify upstream signals inhibiting mTOR signaling in placentas of obese dams, we studied AMPK phosphorylation, which is as a measure of cellular energy status. Activation of AMPK is a known negative regulator of mTOR signaling (Laplante and Sabatini 2012). However, AMPK phosphorylation did not differ significantly between the two groups, offering no explanation of the reduced mTOR signaling in the obese group.

Circulating levels of insulin are markedly increased in the obese dams (Samuelsson et al. 2008). However, the activity of the placental insulin signaling pathway was not altered, consistent with the possibility that the placenta of obese dams develops insulin resistance. Alterations in total protein expression of components of the insulin signaling pathway are known to modulate insulin responsiveness. For instance, it has been shown that an altered protein expression of GRB2 (Liu et al. 2009) and PI3K p85 (Barbour et al. 2005) is associated with changes in insulin sensitivity. In the placentas from the obese dams there was a trend toward reduced GRB2 expression and in other tissues decreased GRB2 expression is associated with increased insulin sensitivity (Liu et al. 2009). However, despite higher maternal insulin levels (Samuelsson et al. 2008) and reduced GRB2, there was no difference in activation of GRB2's downstream target ERK, supporting the idea that in obese dams the placenta develops insulin resistance.

Activation of inflammatory pathways by tumor necrosis factor‐alpha increases mTOR signaling activity (Lee et al. 2007), demonstrating that inflammatory stimuli can alter the activity of the mTOR signaling pathway. Some observations suggest that maternal obesity is associated with placental inflammation, evident by increased infiltration of macrophages in the placental tissue (Challier et al. 2008); however, conflicting findings have been reported (Roberts et al. 2011). Yet, maternal obesity did not affect placental inflammation in our model, and therefore inflammatory signals are unlikely to be responsible for the altered mTOR activity.

Activity of the mTOR pathway can be inhibited by glucocorticoids through increased expression of the protein: regulated in development and DNA damage responses 1 (REDD1) (Wang et al. 2006). In sheep, obesity increases maternal circulating levels of cortisol (Ford et al. 2009). In our obese dams, the corticosterone levels were slightly, but not significantly, higher. However, numerous signals are known to inhibit mTOR signaling and we speculate that signals not directly investigated in this study may lead to the reduced mTOR activity. For example, hypoxia is a known negative regulator of mTOR activity through increased expression of REDD1 leading to activation of TSC2, which is an upstream inhibitor of mTOR (Laplante and Sabatini 2012). Feeding experimental animals a high‐fat, high‐sugar diet may cause a reduction in placental blood flow (Frias et al. 2011), potentially leading to hypoxia or suboptimal oxygenation of the placenta. However, the effect of maternal obesity on placental blood flow in our mouse model is unknown.

Given the well‐established role of mTOR as a positive regulator of placental amino acid transport, the inhibition of placental mTOR in our diet‐induced model of maternal obesity may decrease placental amino acid transport capacity. The decreased placental efficiency, as evidenced by the lower fetal/placental weight ratio in maternal obesity, is consistent with this hypothesis. The activity of placental mTOR is regulated by numerous of upstream signals and we speculate that in this murine model inhibitory signals predominate over stimulatory signals.

## Conflict of Interest

None declared.
